# An overview on glycation: molecular mechanisms, impact on proteins, pathogenesis, and inhibition

**DOI:** 10.1007/s12551-024-01188-4

**Published:** 2024-04-12

**Authors:** Ana Belén Uceda, Laura Mariño, Rodrigo Casasnovas, Miquel Adrover

**Affiliations:** Departament de Química, Universitat de Les Illes Balears, Health Research Institute of the Balearic Islands (IdISBa), Ctra. Valldemossa Km 7.5, 07122 Palma, Spain

**Keywords:** Glycation, Protein structure, Protein function, Protein aggregation, Diabetic-related diseases, Glycation inhibitors

## Abstract

The formation of a heterogeneous set of advanced glycation end products (AGEs) is the final outcome of a non-enzymatic process that occurs in vivo on long-life biomolecules. This process, known as glycation, starts with the reaction between reducing sugars, or their autoxidation products, with the amino groups of proteins, DNA, or lipids, thus gaining relevance under hyperglycemic conditions. Once AGEs are formed, they might affect the biological function of the biomacromolecule and, therefore, induce the development of pathophysiological events. In fact, the accumulation of AGEs has been pointed as a triggering factor of obesity, diabetes-related diseases, coronary artery disease, neurological disorders, or chronic renal failure, among others. Given the deleterious consequences of glycation, evolution has designed endogenous mechanisms to undo glycation or to prevent it. In addition, many exogenous molecules have also emerged as powerful glycation inhibitors. This review aims to provide an overview on what glycation is. It starts by explaining the similarities and differences between glycation and glycosylation. Then, it describes in detail the molecular mechanism underlying glycation reactions, and the bio-molecular targets with higher propensity to be glycated. Next, it discusses the precise effects of glycation on protein structure, function, and aggregation, and how computational chemistry has provided insights on these aspects. Finally, it reports the most prevalent diseases induced by glycation, and the endogenous mechanisms and the current therapeutic interventions against it.

## Introduction

In 1912, Louis-Camille Maillard first described the formation of a set of brownish products as a result of the reaction between sugars and amino acids (Maillard 1912). At that moment, *Maillard chemistry* was born, which tries to understand the complex network of reactions occurring when mixing amino acids or proteins with hydroxyaldehydes or their oxidation by-products (mainly α-oxo-aldehydes). From 1912 to ~ 1970, most of the scientists working in Maillard’s field focused their efforts on trying to understand how these reactions were able to give flavor and taste to foods and drinks, as their comprehension could help to improve their organoleptic features. Simultaneously, it was also discovered that the Maillard reaction occurred in most industrial processes that involve heating, such as those used within the textile (Trézl et al. 1997; Ohe and Yoshimura, 2014), cosmetic (Fusaro and Rice 2010), or biopharmaceutical industries (Zhang et al. 2008), among others. During this period, it was not even appreciated that Maillard reactions could also occur in living organisms, despite the fact that they also contain proteins/amino acids and sugars. However, in 1968, Samuel Rahbar reported a fast-moving hemoglobin electrophoretic band that was particularly evident in blood samples obtained from diabetic people (Rahbar 1968). He had discovered glucose-modified hemoglobin (Hb1A), which nowadays is widely used as glycemic control in diabetic people, and consequently, that the Maillard reaction could also occur in vivo. Although at that moment Dr. Rahbar was not aware of it, his discovery was an incredible breakthrough, as it constituted the foundational proof that Maillard reaction does occur in vivo, and it is associated with the development of most of the diabetic-related diseases (Thorpe and Baynes 1996).

Decades of investigation have proved that, in the body, glucose and its oxidation by-products irreversibly react with the amino groups of intra- and extracellular long-life proteins, DNA, and lipids. When this set of Maillard reactions takes place in vivo, they are known as *non-enzymatic glycosylation* or simply *glycation*, and they lead to the formation of a heterogeneous set of compounds known as *advanced glycation end-products* (AGEs). Usually, the formation of these AGEs modifies the chemical nature of protein residues (i.e., Lys and Arg), amino phospholipids (i.e., phosphatidylserine and phosphatidylethanolamine), or deoxyribonucleic acids (mainly guanosine (Jaramillo et al. 2017; Dutta et al. 2006)) on which they are formed. Consequently, this might have dramatic consequences on the intra- and/or intermolecular interaction pattern of the biomacromolecule, and it might lead to macromolecular misfolding, to aggregation, and, as appreciated recently, to the development of glycation-related diseases (mainly diabetes-related diseases).

Although it has been recently suggested that low level of glycation could have a crucial role to cell survival and homeostasis (Trujillo and Galligan 2023), there are not experimental evidences supporting this idea. Consequently, in this review, we aim at giving to the reader an overall view of what is currently known about glycation and its pathological role. We first explain the similarities and differences between *glycation* and *glycosylation* before going on to better explain the molecular mechanisms underlying glycation reactions. We then describe the most harmful glycating species and their intra- and extracellular bio-molecular targets. Afterwards, we clarify the precise effect of AGEs on protein structure and function, and then explain how computational chemistry can help to get meaningful insights on these effects. We next discuss whether glycation stimulates or inhibits protein aggregation and deposition, and how evolution has designed molecular mechanisms to protect cells against glycation. Finally, we comment on the most prevalent diseases related to glycation and the current therapeutic interventions against it.

### Protein glycation versus protein glycosylation

Most protein scientists are perfectly aware of what *protein glycosylation* is. Nevertheless, we have noticed that very few of them know the chemistry behind *protein glycation* and the pathophysiological consequences of this process. The only similarity between glycosylation and glycation is that both describe processes through which carbohydrates modify proteins. Yet, their molecular mechanisms and their functions in physiology and pathology are very different.

*Glycosylation* is a well-controlled process that is part of the protein biosynthesis machinery (Ohtsubo and Marth 2006). It encompasses all the post-translational modifications mediated by glycosyltransferases, the enzymes which catalyze the formation of glycosidic bonds between a target protein and specific polysaccharides, thus assembling glycoproteins. Glycosylation mainly occurs in the endoplasmic reticulum and Golgi apparatus of the secretory pathway (Hang et al., 2015). The specific types of glycosylation are named according to the last sugar of the polysaccharide chain. Hence, we can find sialylation, fucosylation, galactosylation, multibranching of glycans (bi-, tri-, tetra-, penta-antennary), etc. (Blomme et al. 2009). In fact, the number of glycan determinants comprising the human glycome is not exactly known, but it has been estimated to exceed more than 3000 (Cummings 2009). Numerous enzymes, molecular chaperones, and cell transport proteins are involved in the biosynthesis of these polysaccharides and in the formation of specific glycosidic bonds with target proteins. Altogether, these different proteins constitute the cellular glycosylation machinery. The glycans attached to proteins via enzymatic glycosylation can be classified into two types: (i) the *N*-glycans, which are those attached to the amide side chain of Asn residues; and (ii) the *O*-glycans, encompassing those attached to the hydroxyl group of side chains of Ser or Thr residues (Kobata 1992).

**Fig. 1:**
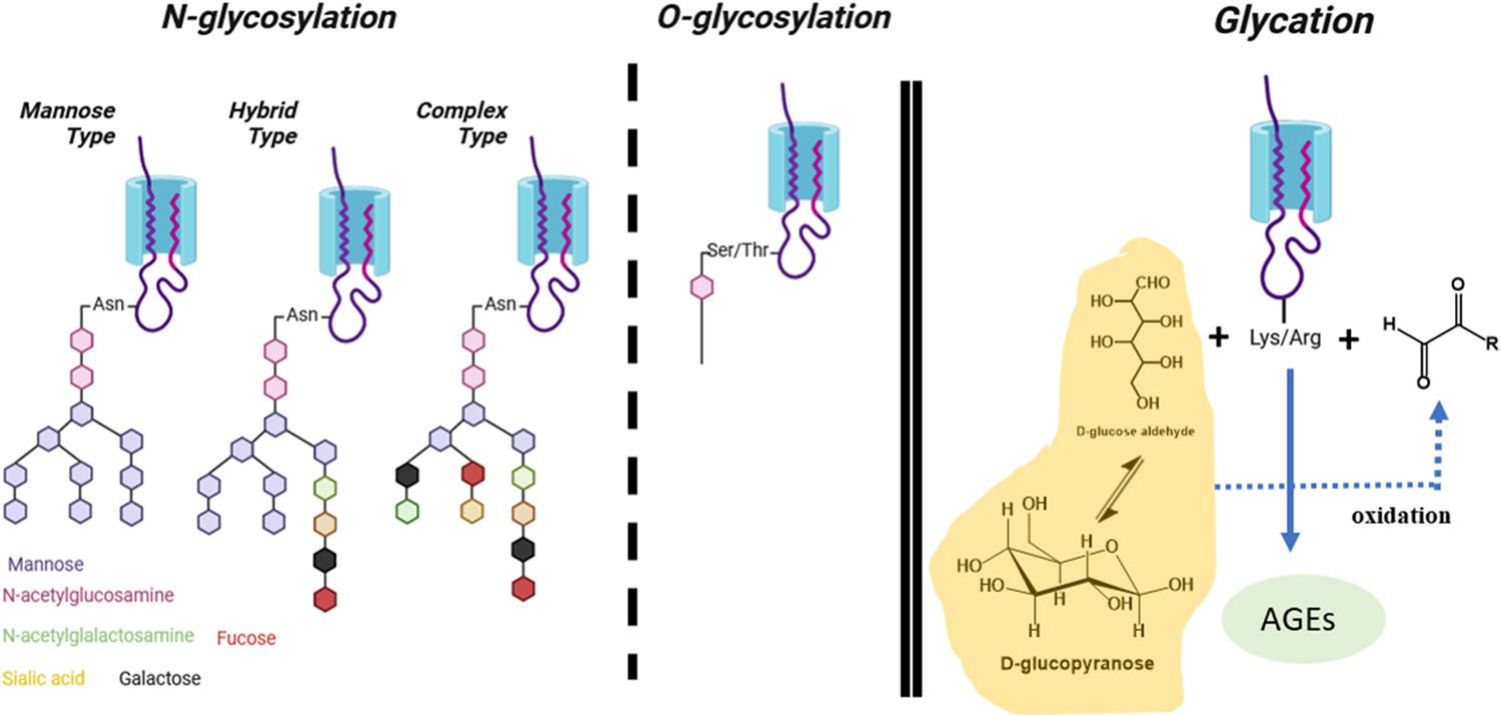
Protein glycosylation (*left*) and protein glycation (*right*). In the protein *glycosylation*, the carbohydrates can be attached either to an Asn (N-glycosylation) or to a Ser/Thr (O-glycosylation) residue. N-Glycosylation can be of three different patterns: (i) high mannose type; (ii) hybrid type; and (iii) complex type. In the O-glycosylation, the first carbohydrate bound to a Ser/Thr residue is a GlcNAc and after it, there is not a clear pattern, as many different types of carbohydrates have been found. Protein *glycation* starts with the reaction of a reducing sugar (mainly glucose) or an α-hydroxyaldehyde (which might come from glucose autoxidation) with the Lys/Arg side chains of long-life proteins. These reactions yield the formation of a heterogeneous set of compounds named as AGEs

The core structure of *N*-linked polysaccharides consists of a complex pentasaccharide composed of two *N*-acetylglucosamines (GlcNAc) and one branched mannose (α-1,3 and α-1,6), which is bound to two additional mannose units. After these two, various types of sugars can incorporate varying numbers of additional molecules (i.e., fucose, mannose, GlcNAc, sialic acid, etc.), although they tend to adopt one of three different patterns: (i) high mannose type (all the following units are mannose); (ii) complex type (a heterogeneous set of carbohydrate moieties); and (iii) hybrid type (one mannose is bound to a polymannose chain and the other is bound to a heterogeneous set of carbohydrate moieties). *N*-Glycosylation plays a crucial role in protein biogenesis and function (Fig. 1). Besides its potential involvement in regulating brain development and function (Handa-Narumi et al. 2018), *N*-glycosylation exerts its important effects by influencing protein folding, cellular localization, stability, and solubility.

In contrast, the only common feature of the *O*-linked polysaccharides is that a GlcNAc is attached to the hydroxyl group of Ser or Thr (Halim et al. 2011). After this, sugar follows a series of core structures capable of forming extended linear or branched polysaccharide backbones (Fig. 1). *O*-Glycosylation occurs in any protein that follows the secretory pathway (Bennett et al. 2012) and it is highly abundant in mucins (Steentoft et al. 2013). This type of glycosylation plays a crucial role in numerous biological and pathological processes, including tumor growth and tumor progression.

In any case, once a protein is *N-* or *O-*glycosylated, the polysaccharide moiety modifies its structure, function, and aggregation propensity, thus having a remarkable impact on its biological activity. Given their biological relevance for the correct cellular function, alterations in protein glycosylation are associated with the pathogenesis of many diseases such as cancer, infections, or autoimmune disorders (Spiro 2002).

While polysaccharides are the species that enzymatic *glycosylation* binds to proteins, monomeric glucose is responsible for triggering, directly (in the extracellular space) or indirectly (in the intracellular space), the random and aberrant *glycation* process (Shin et al. 2023). Glucose is the main source of energy for mammals. It is absorbed through the thin intestine, via a trans-epithelial transport system (SGLT-1 and SGLT-2), and is then directed to the liver where it is converted either into glycogen (glycogenesis) or into lipids (lipogenesis) (Han et al. 2016). Plasma glucose is taken up by cells through an active mechanism, which is activated by insulin when needed (Petersen and Shulman 2018). Once in the cytoplasm, glycolysis starts to produce ATP (Guo et al. 2012). There are several pathological conditions that cause a decrease in glycolysis and an increase in plasma glucose. The most important one is diabetes mellitus (DM), an endocrine disorder characterized by the absence of insulin (type 1 DM; DM1) or the inability of cells to respond to it (type 2 DM; DM2). The latter raises the normal fasting glucose levels (≤ 99 mg/dL) up to values ≥ 126 mg/dL (Janghorbani et al. 2007; Sakran et al. 2022).

In solution, glucose displays equilibria between a tiny proportion of lineal aldehydic form (0.002%) and different cyclic hemiacetal isomeric forms (Dworkin and Miller 2000). Albeit the percentage of the lineal form is almost negligible under physiological conditions, its remarkable electrophilicity makes it capable of aberrantly reacting with the nucleophilic groups of long-lived biomacromolecules, such as the amino phospholipids of the outer leaflet of cell membranes, or the Arg and the Lys side chains of both plasma proteins and transmembrane proteins (Semba et al. 2010). This is how glucose triggers extracellular *glycation*, a complex cascade of oxidative reactions that end with the formation of AGEs, which can impact the structure of targeted biomacromolecules and, thereby, alter biological function and promote pathological problems (Fig. 1). The glycating effect of glucose is not limited to the extracellular space. During intracellular glycolysis, a set of highly reactive carbonyl compounds is produced with the most important one being methylglyoxal (MG), an α-oxoaldehyde, whose cellular concentration is approximately 300 μM (Thornalley 1996; Chaplen et al. 1998). Due to its high nucleophilicity and toxicity, evolution has designed enzymatic mechanisms to regulate the levels of intracellular MG, such as the glyoxalase system (Glo-1 and Glo-2). However, both enzymes are downregulated under DM (Aragonès et al. 2021), and therefore, the levels of MG in people suffering from DM are two to five times higher than those in healthy people. Consequently, these intracellular upregulated MG levels promote glycation reactions on the long-life cytoplasmatic proteins, on the inner leaflet of cell membranes, on the outer leaflet of cell organelles, and on DNA (Allaman et al. 2015; Schalkwijk and Stehouwer 2020). All this indicates that glycation is a random and harmful process (stimulated by hyperglycemia and aging) that occurs in the intra- and in the extracellular space, which yields the accumulation of AGEs that are involved in the development of most of DM-related diseases and some aging-related diseases.

### Molecular mechanisms underlying protein glycation

**Fig. 2:**
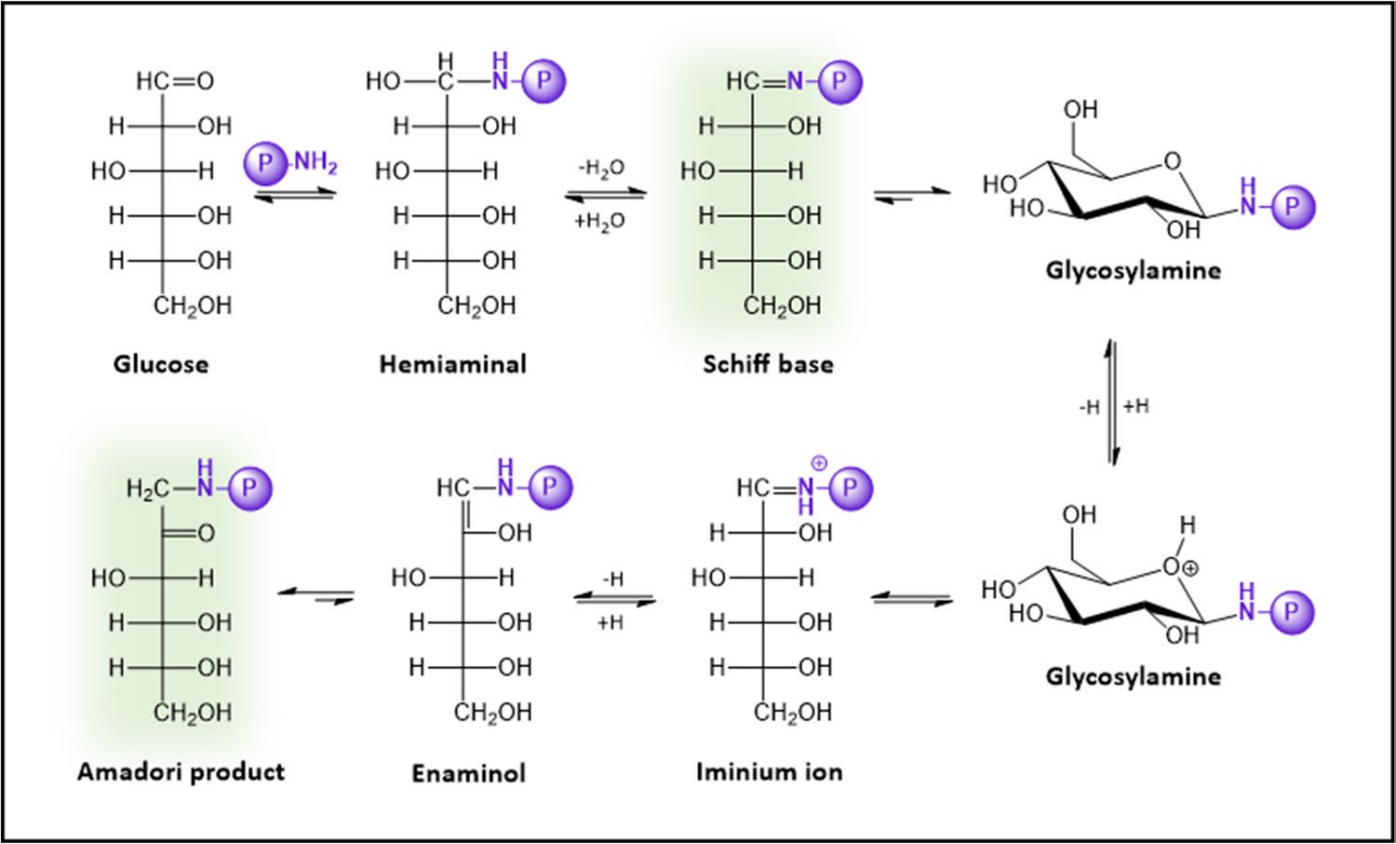
Molecular mechanism corresponding to the formation of a Schiff base from the reaction between the primary amine of a protein (P) and the carbonyl group of glucose (*top*). Once the Schiff base is formed, it rearranges to form an Amadori product (*bottom*)

**Fig. 3:**
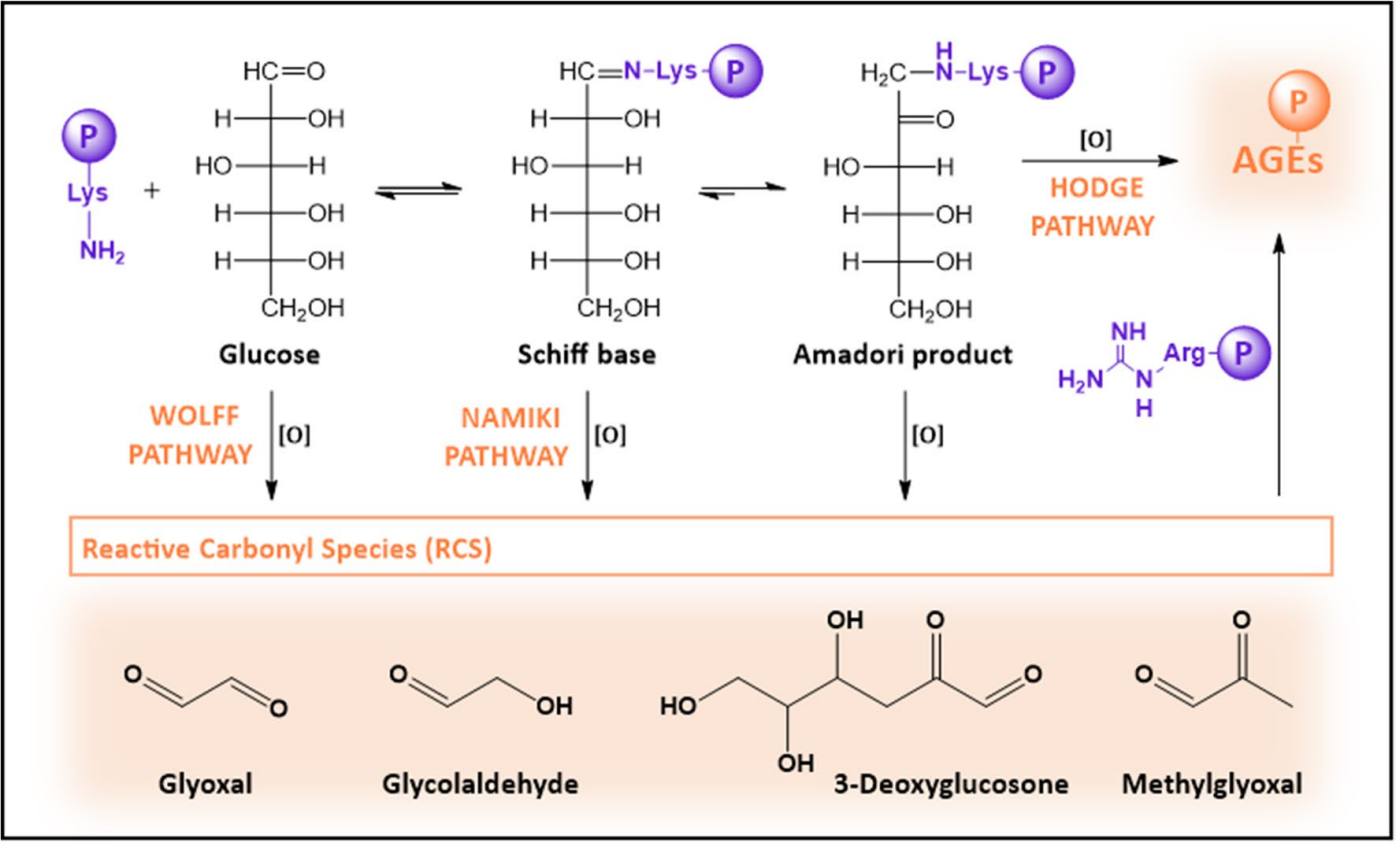
Scheme of different pathways of AGE formation. The reaction between the ε-amino group of Lys and the carbonyl group of glucose forms a Schiff base, which rearranges to form an Amadori product. Some Amadori products are converted to AGEs by the Hodge pathway, and others are oxidized and cleaved to form RCS. These RCS are also generated by the Wolff and Namiki pathways from glucose and Schiff base, respectively. These RCS can further react with proteins to generate AGEs

The molecular mechanism underlying *the extracellular glycation* process starts with the reaction between a primary amino group of a protein (i.e., the N-terminal α-amino group or the ε-amino group of Lys residues), and the carbonyl group of glucose. This leads to the formation of a Schiff base (Fig. 2) (Cho et al. 2007), which displays equilibria between a minor open-chain aldimine and a major and more stable glycosylamine ring (Irvine and Campbell 1922). This aldimine is highly unstable and, thus, it rapidly undergoes an Amadori rearrangement to form a stable ketoamine, known as an Amadori product (Cho et al. 2007; Yaylayan and Huyghues-Despointes 1994). The Amadori rearrangement normally involves an acid-catalyzed ring opening of glycosylamine to give an iminium ion, which then undergoes deprotonation to form a 1,2-enaminol in equilibrium with the Amadori product (Cho et al. 2007). The formation of a Schiff base is relatively fast and highly reversible, whereas the formation of an Amadori product is slower, although thermodynamically more favored (*k*_formation_ 14.2 × 10^−6^ s^−1^, *k*_dissociation_ 1.7 × 10^−6^ s^−1^), so they tend to accumulate (Yaylayan and Huyghues-Despointes 1994). However, with time and under appropriate conditions, Amadori products can undergo different reactions (through the Hodge pathway) to irreversibly produce a heterogeneous set of AGEs (Fig. 3) (Yaylayan and Huyghues-Despointes 1994).

The formation of Schiff bases, Amadori compounds, and their final evolution to AGEs constitutes the backbone of the extracellular glycation process. However, all this becomes much more complex as sugar moieties can undergo multiple fragmentation reactions (i.e., dehydration or oxidation), which might occur prior to their attachment to proteins, or alternatively once the Schiff base or the Amadori product is formed. These parallel reactions, which constitute the Wolff and Namiki pathways, produce highly reactive carbonyl species (RCS), such as 3-deoxyglucosone (3-DG) (Anet 1960), MG, or glyoxal (GO) (Thornalley et al. 1999) (Fig. 3). These RCS can further modify Lys and Cys side chains (Zeng and Davies 2005), although their main targets are Arg (Lo et al. 1994), whose reaction mainly yields hydroimidazolone-like AGEs (MG-Hs) (Ahmed et al. 2003). Consequently, these RCS increase the diversity of the AGEs, and propagate the damage initiated by glucose (Nass et al. 2007). In fact, more than 20 different AGEs have been identified in human blood, tissues, and food (Perrone et al. 2020), and depending on their origin, structures, and properties, they can be categorized into several groups (Table 1) (Twarda-Clapa et al. 2022).

**Table 1:** Criteria for classification of the different AGEs according to source, precursor, and ability to emit fluorescence)

CLASSIFICATION OF AGEs	Source	Endogenous
		Exogenous
	Precursor	Glucose-derived AGE
		GLA-derived AGE
		MG-derived AGE
		GO-derived AGE
		3-DG-derived AGE
	Chemical structure and ability to emit fluorescence	Crosslinked and fluorescent
		Crosslinked and non-fluorescent
		Non-crosslinked and fluorescent
		Non-crosslinked and non-fluorescent

**Fig. 4:**
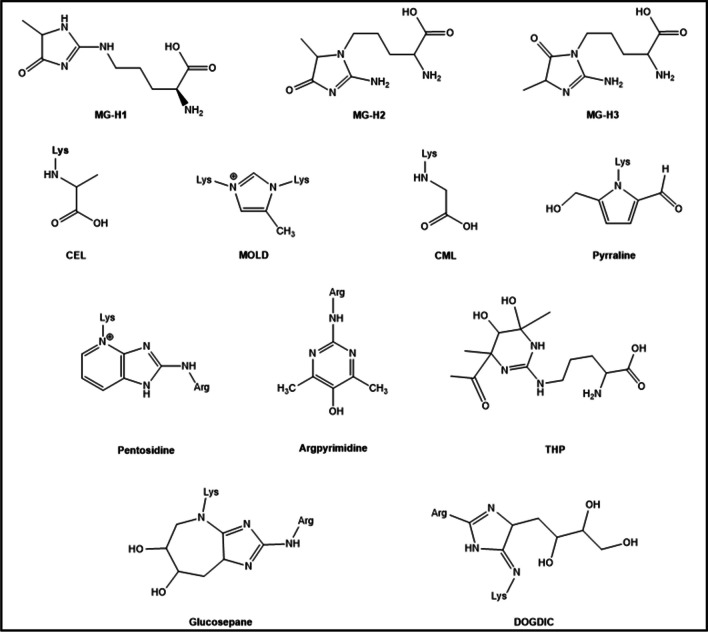
Chemical structure of the more relevant characterized AGEs: N^δ^-(5-methyl-4-imidazolon-2-yl)-l-ornithine (MG-H1), 2-amino-5-(2-amino-5-hydro-5-methyl-4-imidazolon-1-yl)pentanoic acid (MG-H2), 2-amino-5-(2-amino-4-hydro-4-methyl-5-imidazolon-1-yl)pentanoic acid (MG-H3), N^ε^-carboxyethyl-lysine (CEL), N,N(-di(N^ε^-lysino))-4-methyl-imidazolium (MOLD), N^ε^-carboxymethyl-lysine (CML), pyrraline, pentosidine, argpyrimidine, and N.^δ^-(4-carboxy-4,6-dimethyl-5,6-dihydroxy-1,4,5,6-tetrahydropyrimidine-2-yl)-l-ornithine (THP), glucosepane, and N(6)-(2-((4-ammonio-5-oxido-5-oxopentyl)amino)-5-(2,3,4-trihydroxybutyl)3,5-dihydro-4H-imidazol-4-ylidene)lysinate (DOGDIC)

Since glucose rapidly undergoes glycolysis in the cytoplasm, the *main glycating compounds in the intracellular space* are the upregulated RCS produced as side products of glycolysis (Takeuchi et al. 2021). Although GO and 3-DG display a high glycation potential, the main glycating activity in the cytoplasm is attributed to MG as a result of its high concentration and its high reactivity (20 × 10^3^ times higher than that of glucose (Thornalley 2005)). When MG reacts with Lys, it forms N^ε^-(1-carboxyethyl)lysine (CEL) (Ahmed et al. 1997) and 1,3-di(N^ε^-lysino)-4-methyl-imidazolium (MOLD), which is a crosslinking AGE (Brinkmann et al. 1998). When it reacts with Arg, it forms N^δ^-(4-carboxy-4,6-dimethyl-5,6-di-hydroxy-1,4,5,6-tetra-hydropyrimidine-2-yl)ornithine (THP) (Oya et al. 2000) and argpyrimidine (Shipanova et al. 1997), but the major adducts found in vivo are the MG-Hs (Ahmed et al. 2003). They are formed as three structural isomers: N^δ^-(5-hydro-5-methyl-4-imidazolon-2-yl)-ornithine (MG-H1), 2-amino-5-(2-amino-5-hydro-5-methyl-4-imidazolon-1-yl)pentanoic acid (MG-H2), and 2-amino-5-(2-amino-4-hydro-4-methyl-5-imidazolon-1-yl)pentanoic acid (MG-H3) (Fig. 4).

Although we have explained the basic constituents involved in the glycation mechanism, its reaction pathway shows a high dependence on environmental conditions, such as the reactivity and the concentration of the glycating agent, the reactivity of the glycating target, the presence of catalysts (i.e., metals, oxygen, or buffer ions), the temperature, or the pH, among others (Johansen et al. 2006).

**Fig. 5:**
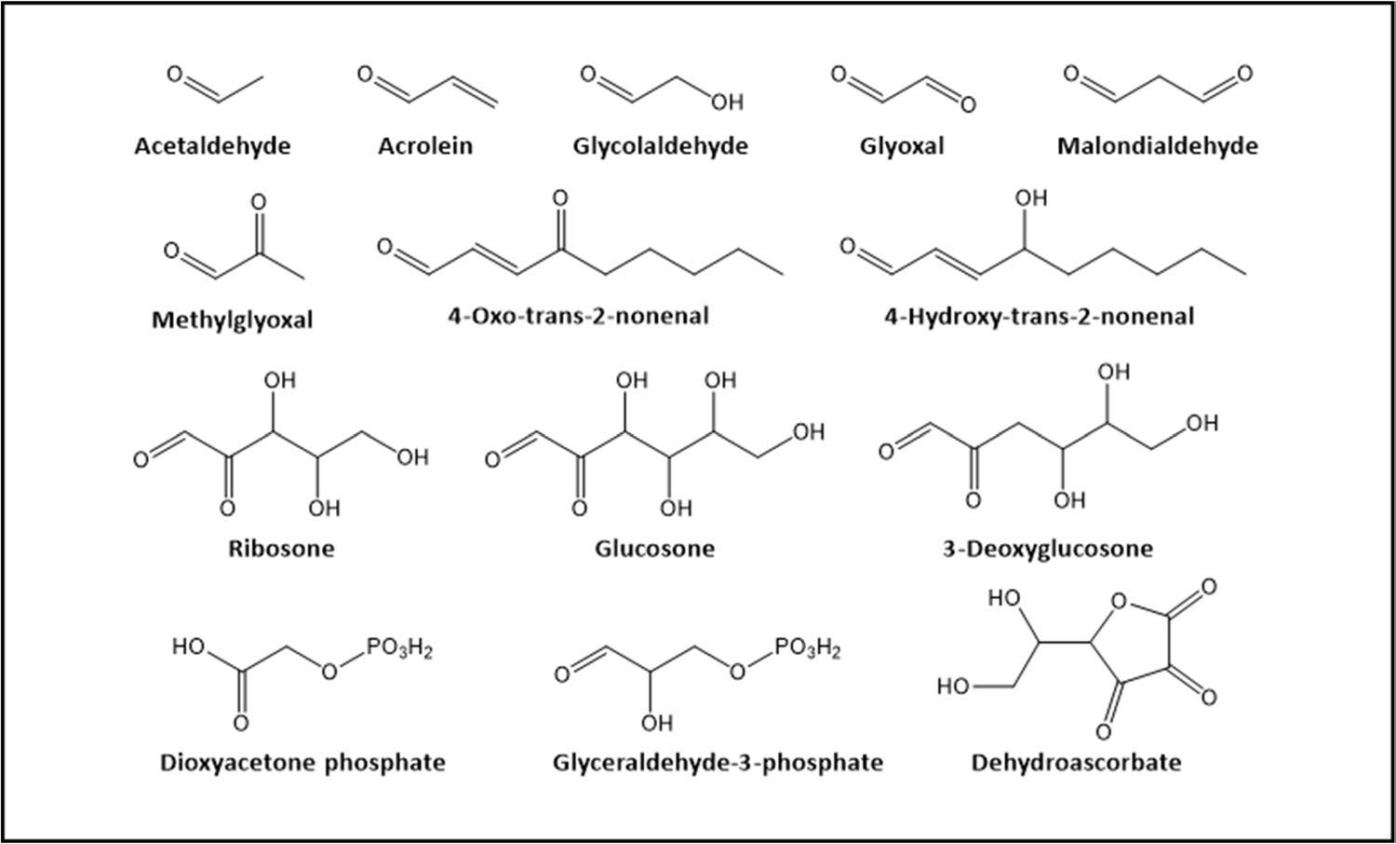
Chemical structure of the most common biological reactive carbonyl species

Among the entire set of *glycating agents*, RCS are the most harmful ones. The presence of an α-oxo-aldehyde group in their structure confers to them a high reactivity, with the unsaturated ones showing more reactivity than their saturated counterparts. In total, more than 20 different RCS have been found in living organisms (Fig. 5) (Niki 2009). Endogenous RCS come from amino acid oxidation, lipid peroxidation, glycolysis, or glycation, whereas exogenous RCS come from pollutants, cigarette smoke, food additives, or browned food (Semchyshyn 2014). In any case, their high reactivity makes them responsible for ~ 65% of the total cellular glycation events, and their chemical nature determines the type of AGEs formed on the targeted biomolecule, and therefore, the consequences that they have on their structure and function. However, they are only toxic when there is a dysregulation of the mechanisms designed to control their production and/or elimination, an event known as carbonyl stress. However, when RCS are found in low steady-state concentrations, they play important roles in immune response, as regulators of gene expression and as messengers of cellular signaling (Uchida 2000; Forman et al. 2008; Niki 2009).

In addition to RCS, reducing sugars can induce glycation (mainly in the extracellular space), although not all of them display the same reactivity. The larger the percentage of the open-chain form, the more reactive is the sugar (Monnier 1990), with the population extent of the open chain being inversely proportional to the number of carbon atoms in the molecule. Aldoses react more rapidly than ketoses (Bunn and Higgins 1981), because of the greatest electrophilic character of their carbonyl groups. Charged sugars, such as phosphorylated ones, are more reactive than their uncharged counterparts due to the presence of electrostatic interactions (Bunn and Higgins 1981). Accordingly, the glycating ability of reducing sugars increases as d-glucose < d-mannose < d-galactose < d-fructose < d-arabinose < d-ribose (Monnier 1990).

With regard to proteins, the ε-amino group of Lys and the guanidinium group of Arg are the main protein *glycation targets* in vivo. Additionally, RCS can also modify the thiol group of Cys residues. However, this only occurs under reducing environments, such as the cytoplasm (Thorpe and Baynes 2003). Besides the amino acid type, there are other factors that affect the glycation propensity of each residue (i.e., neighbor side chains, salt-bridge interactions, or steric disposition) (Johansen et al. 2006). The rate of Schiff base formation is affected by both the pK_a_s of the amino groups and their accessibility to the glycating agent. Meanwhile, the rate of Amadori rearrangement is accelerated by local acid–base catalysis driven by carboxyl groups of acidic residues, ε-amino groups of Lys residues, and His imidazole rings (Johansen et al. 2006; Takahashi 2015).

The extent of protein glycation is also strongly dependent *on the environmental conditions* (i.e., the presence of catalysts, the temperature, or the pH) and on the half-life of the target protein. The formation of Schiff bases is favored by the presence of biological buffers such as phosphate or bicarbonate (Johansen et al. 2006), which can act either as acid–base catalysts or as promotors of the reaction, as they can stabilize the reactive open-chain form of glucose (Burton and McWeeny 1963). Changes in pH and/or temperature can also influence the extent of Schiff base formation since the proportion of sugars in their reactive open-chain form is pH-dependent, as it does the protonation state of target residues (mainly Lys), which must be deprotonated to start the glycation process (Martins and van Boekel 2005). On the other hand, the amount of Amadori product correlates with glucose concentration, but also with the half-life of the modified protein. Hence, the Amadori content in proteins depends on whether the survival time of the target protein is longer than the time required to reach the equilibrium (~ 28 days) (Bucala et al. 1991).

### Othermolecular targets of glycation

In addition to proteins, glycation can also occur on nucleic acids and amino phospholipids, as both possess free amino groups. Nucleic acids can be modified by reducing sugars and RCS to form DNA-AGEs, which impact DNA structure and functionality (Ahmad et al. 2011; Ashraf et al. 2016; Bagherzadeh-Yazdi et al. 2020). N^2^-Carboxyethyl-2′-deoxyguanosine is the most common AGE found in DNA and it has been described as a potential biomarker of chronic hyperglycemia (Jaramillo et al. 2017). Glycation of nucleic acids can lead to mutations, strand breakage, and reduced gene expression (Pischetsrieder et al. 1999; Wuenschell et al. 2010; Ashraf et al. 2012). Additionally, glycation of DNA contributes to aging and to the pathogenesis of diseases such as DM, cancer, inflammation, and neurodegeneration (Voulgaridou et al. 2011; Rehman et al 2022).

Amino phospholipids, such as phosphatidylethanolamine and phosphatidylserine, both present in mammal cell membranes, have been found to become abnormally glycated under hyperglycemic conditions (Nakagawa et al. 2005). This may lead to the impairment of the integrity and functionality of cells (Oak et al. 2003). Hence, the formation and accumulation of glycated amino phospholipids have been associated to the pathogenesis of hyperglycemia in diabetic complications such as angiogenesis, atherosclerosis, or inflammation (Oak et al. 2003; Basta et al. 2004; Miyazawa et al. 2012) and age-related dysfunctions of different tissues (Fournet et al. 2018; Uceda et al. 2024).

### Effect of glycation on protein structure: a field full of fog

As previously mentioned, the glycation of biomolecules seems to be one of the major factors behind the development of DM-related diseases. However, it is not entirely clear how this occurs at molecular level. In proteins, AGEs are mostly formed on Lys and Arg side chains, two basic amino acids holding a positively charged side chain at physiological pH. In most cases, the formation of these AGEs on Lys and/or Arg implies the depletion of their positive charges, thus turning these residues into neutral, zwitterionic, or even anionic residues. Therefore, it was thought that if a long-life protein has a considerable number of basic amino acids in its sequence, their glycation might have a dramatic impact on (i) its electrostatic potential pattern, (ii) its folding, (iii) its aggregation tendency, and (iv) its function.

For a long time, it was assumed that glycation had a direct chaotropic effect on the protein structure, which resulted in hydrophobic exposure (Wei et al. 2009; Roy et al. 2010; Fazili and Naeem 2013; Bakhti et al. 2007; Bathaie et al. 2011). However, this assertion was always made from the data obtained using low-resolution techniques, such as circular dichroism (CD), UV–Vis, and fluorescence spectroscopies. The fluorescence red shift and/or the reduction in the protein-Trp quantum yield during glycation have typically been ascribed to protein unfolding events. Such observations have suggested that glycation of lysozyme (Ghosh et al. 2013), cystatin (Mustafa and Bano 2016), ribonuclease A (Dinda et al. 2015), and albumin (Sadowska-Bartosz et al. 2014) by glucose, fructose, or ribose affects their tertiary structure. MG was suggested to unfold myoglobin (Banerjee et al 2016), as does GO with hemoglobin (Iram et al. 2013) or ribose on albumin (Wei et al. 2009), on glucose oxidase (Khan et al. 2012), or on phytocystatin (Ahmed et al. 2017). Fluorescent data has suggest that glucose has a chaotropic effect on albumin (Sattarahmady et al. 2007) and on immunoglobulin G (Ahmad et al. 2012), while reduction in the Trp quantum yield of α- and γ-crystallin associated to their incubation with fructose allowed to conclude that this carbohydrate is able to structurally modify both proteins (Lüthra and Balasubramanian 1993).

The possible effect of glycation on protein structure was additionally assessed using extrinsic fluorescent dyes. 8-Anilino-1-naphthalenesulfonic acid (ANS) (λ_exc_ ~ 380 nm; λ_em_ ~ 400–600 nm) is a highly sensitive dye with respect to polarity since it displays a blue shift of its fluorescence emission maximum and an increase of quantum yield upon binding to protein hydrophobic pockets (Stryer 1965). ANS was used to suggest that glycation induces molten globule (partially folded species) formation in phytocystatin glycated with ribose (Ahmed et al. 2017) and during the glycation of hemoglobin with GO (Iram et al. 2013).

Although such results clearly point towards a glycation-induced unfolding effect, these findings require bolstering with further studies based on medium- or high-resolution techniques. As glycation is a non-enzymatic process, when it randomly occurs on a protein, it generates a heterogeneous set of molecules with different glycation degrees and different types of AGEs formed at the same “hot-spot.” Such heterogeneity has generally precluded the widespread use of crystallography for the study of glycated proteins. However, in the Protein Data Bank (PDB), there is a single crystal structure on a glycated protein, corresponding to hemoglobin glycated with fructose (PDB: 3B75), which was deposited in 2007. Its structure overlaps fairly well with that corresponding to native hemoglobin, thus proving that glycation-mediated fructose does not necessarily induce protein unfolding nor the formation of molten globules.

To clarify the exact effect of glycation on protein structure, NMR spectroscopy has been used to provide structural information at residue level. NMR was applied to study how glycation mediated by ribose and glycolaldehyde (GLA) affected the structure of hen egg white lysozyme (HEWL), a well-studied model of protein folding (Mariño et al. 2017; Adrover et al. 2014). Although it was clear that ribose and GLA were able to form AGEs on HEWL, NMR unequivocally proved that their formation was not sufficient to modify the native tertiary structure of HEWL. The maintenance of the native-like structure of a protein upon glycation was not only recorded for HEWL, as it was also observed for cytochrome C (Oliveira et al. 2013) and insulin (Oliveira et al. 2011). Whether or not glycation was able to impact the α-helical conformation of a 15-residue model peptide holding a Lys at the middle of its sequence has also been studied using a combination of NMR, CD, and molecular dynamics (MD) simulations. This combined approach indicated that neither ribose nor MG were able to disrupt the secondary structure of the peptide (Mariño et al. 2019). In summary, all of the experimental data obtained from the use of NMR and X-ray diffraction prove that glycation is not able to alter the secondary nor the tertiary structure of a glycated protein.

In principle, logical reasoning should expect these results, since Lys and Arg are usually solvent-exposed residues located at the protein surface and normally, lacking of important long-range contacts defining protein tertiary structure. Consequently, and unless Lys/Arg participate in long-range salt bride interactions, we may assume that glycation should not affect the protein intramolecular interaction pattern assembling the protein structure. These results beg the question, where do the discrepancies between the interpretation of the data obtained by fluorescence spectroscopy and those obtained from NMR spectroscopy and X-ray diffraction originate? In an attempt to answer this question, our group studied the possible quenching effect of AGEs formed on isolated Lys and Arg amino acids on the protein intrinsic fluorescence and on the ANS fluorescence (Adrover et al. 2014). AGEs formed on both amino acids, when using ribose or GLA, were able to quench the intrinsic fluorescence of HEWL, as well as the fluorescence emission of ANS. We found that the formation of AGEs on a protein exerts a quenching effect on its intrinsic fluorescence (Adrover et al. 2014). Unfortunately, the literature is full of manuscripts misinterpreting fluorescence data derived from the study of glycated proteins, just because they do not take into account this plausible quenching effect, thus providing wrong data interpretation and confusing results. Consequently, we suggest that fluorescence data alone should not be used to derive structural considerations when studying glycation reactions or glycated proteins.

### Effect of glycation on protein function

Independent of any potential chaotropic effect that glycation could have on the protein structure, it is highly likely that AGE formation disrupts protein function. The replacement of cationic residues by AGEs possessing a different chemical nature (i.e., anionic, neutral, zwitterionic, hydrophobic or those involving intramolecular covalent crosslinking; (Fig. 4)) could (i) generate new surface hydrophobic patches that stimulate aggregation (Adrover et al. 2014); (ii) modify the equilibria between the folded species and any partially folded/unfolded counterparts; (iii) weaken or strengthen protein interactions with its biological counterparts/binding partners; or (iv) change the enzymatic activity decreasing the *k*_cat_ and/or increasing the K_M_. As a result, it has been reported that the glycation of human and bovine serum albumin result in an altering of their protein function and cytotoxicity (Anguizola et al. 2013; Baraka-Vidot et al. 2013; Byun et al. 2012; Shaklai et al. 1984). Glycation turns cytochrome c into an inducer of apoptosis (Sharma et al. 2019) and can also impair the enzymatic functions of high-density lipoprotein paraoxonase (Hedrick et al. 2000) and hemoglobin peroxidase activities (Bose et al. 2013; Ghoshmoulick et al. 2007). In contrast, myoglobin glycated with glucose seems to enhance hemoglobin peroxidase activity (Roy et al., 2010) yet induce an antioxidative activity upon glycation with fructose (Bhattacherjee and Chakraborti 2011). In addition, it seems that MG enhances the chaperone activity of proteins like α-crystallin and Hsp27, but the opposite effect occurs when α-crystallin is incubated with fructose, glucose, or ascorbate (Kumar et al. 2004; Mukherjee et al. 2023; Nagaraj et al. 2003). The glycation of HEWL with ribose or GLA was shown to result in a reduction of its enzymatic activity (Mariño et al. 2017; Adrover et al. 2014), with this general effect also seen for alkaline phosphatase (McCarthy et al. 1998) as well as for many other enzymes. Additionally, protein function can also be altered by impaired protein–ligand interactions resulting from AGE formation, protein charge modification, or conformational changes. For example, glycation of human erythrocyte glutathione peroxidase has been shown to alter its stability and heat resistance, thus resulting in a reduction of its affinity for the substrate (Suravajjala et al. 2013).

Furthermore, glycation has been proved to contribute to oxidative stress, as AGEs are known to generate reactive oxygen species (ROS). Consequently, under hyperglycemic and oxidative stress conditions, proteins can be damaged. This can lead to cellular dysfunction and inflammation and also to protein dysfunction (Bansal et al. 2012; Mercado-Uribe et al. 2020; Nowotny et al. 2015; Wang et al. 2015). Moreover, glycation is also able to hamper the antioxidant capacity of certain long-lived proteins, thus affecting the cellular antioxidant machinery. For instance, AGE formation on α-synuclein has been proved to deplete its ability to prevent the formation of ROS (Martínez-Orozco et al. 2019).

In summary, the effects of glycation on protein function are complex and can vary depending on the specific protein, the extent of glycation, and the cellular environment.

### Computational chemistry: a promising tool to unravel the precise effect of glycation on protein structure and biological function

Most of what we currently know about the effect of glycation on protein structure and function has been achieved using experimental methodology. Although computational chemistry methods allow for the acquisition of information from molecular systems with atomic resolution, there are not many standalone computational studies dedicated to understanding different aspects of glycation.

One of the earliest works was dedicated to understand the effects of glycation on human hemoglobin. Through adsorption isotherms, it was found that glycated hemoglobin exhibits a higher affinity for oxygen than the native form, although having a lower affinity for 2,3-diphosphoglycerate (DPG) (De Rosa et al. 1998). These MD simulations revealed that the carbohydrate moiety of the glycated form occupies the DPG binding site, thus preventing it from adopting one of the two possible binding conformations.

Collagen is a protein that has been extensively studied using computational methods to understand the effect of glycation. In 2014, a collagen model was created to identify potential Lys-Arg cross-linking sites for the formation of glucosepane (the most common AGE found in type I collagen) (Gautieri et al. 2014). MD simulations were used to measure which Lys and Arg remain close to each other enough time to form glucosepane and, hence, to identify the most probable cross-linking sites. Similarly, Collier et al. analyzed the distances between Lys-Arg pairs to identify potential sites for the formation of DOGDIC. Structurally, the formation of DOGDIC did not change the backbone conformation. MD simulations provided the enthalpic variation due to DOGDIC formation and estimated its thermodynamic plausibility. In any case, the sites prone to be targets for glucosepane and DOGDIC (Fig. 4), although they were not the same, were identified as interaction sites between collagen and other proteins such as integrins, proteoglycans, and collagenase, thus providing an explanation for the observed reduced affinity upon glycation (Collier et al. 2015, 2016).

Elastin is another protein of structural importance whose mechanics were found to be altered upon glycation. MD simulations were carried out on tropoelastin under hyperglycemia. Under this condition, three binding sites remain occupied by glucose throughout the simulation, although they were not close to the glycation targets. This binding leads to a local increase in coil conformation and a reduction in the beta-sheet content. Although this study only considered glucose in a cyclic form, the authors hypothesize that an effect of hyperglycaemia is that cyclic glucose exposes glycation targets, thus giving them a higher propensity to be glycated (Yang et al. 2023).

Jeevanandam et al. used MD simulations to study changes in the structure and dynamics of insulin due to glycation. Glycation of R22 to form MG-derived hydroimidazolone (MG-H1) (Fig. 4) caused insulin to adopt a closed and highly stable conformation that reduced the exposure to the solvent of the hydrophobic core. It also decreased the β-sheet content of the monomer and increased the coil content, possibly due to a reduced accessibility to the open conformation. Insulin must undergo a conformational change in the central region of chain-B from open to wide open to bind to the insulin receptor. Therefore, glycation on R22 may prevent insulin from binding to its receptor (Jeevanandam et al. 2023a, b).

The effect of glycation on human serum albumin was also studied using MD simulations (conducted by Sittiwanichai et al. 2023) to examine two different models of glycation. The first contained a Schiff base formed on K195, and in the second one, K195 was modified by an Amadori product. The covalent attachment of both carbohydrate moieties induced a subtle loss in helicity (~ 10%) and an alteration in protein dynamics of the domains I and III. In addition, W84 interacted less with C34, thus becoming less shielded and more reactive. Additionally, the environment of W214 also changed due to glycation. As a result, both Sudlow’s sites I and II, along with fatty acid binding sites, are clearly decreased in size, suggesting a decrease in the transport capacity of albumin due to glycation (Sittiwanichai et al. 2023). In addition, Jeevandam et al. also investigated the effect of MG-H1 formation on R114, R186, R218, R410, and R428, both individually and collectively. MD simulations revealed alterations in the dynamics and accessibility to the Sudlow’s binding sites. In the native state, and in the R410-MG-H1 and R4238-MG-H1 variants, dynamics increased the space between domains I and III, favoring the exposure of Sudlow I. In the R186-MG-H1, R218-MG-H1, and fully glycated forms, the opposite occurred. Only R114-MG-H1 showed alteration in both behaviors. Glycation of R218 and R428 did not modify the interaction between W84 and C34. However, the glycation of R114, R186, and R410, and as well as the total glycation, broke the H-bond between W84 and C34, thus suggesting a glycation-induced increase in the reactivity of C34. Regarding the binding sites, Sudlow I is only partially altered by the glycation of R114 and R410, resulting in a partial opening of the site. For Sudlow II, the modification of different Arg residues strengthened or weakened some interactions between residues that form it, with total glycation causing weakening and complete opening. In summary, although the overall effect on global structure is small, glycation was shown to have significant consequences for the protein’s transport capacity (Jeevanandam et al. 2023a, b). Moreover, Pongprayoon et al. studied the binding of glucose to albumin. They observed that pyranose forms of glucose do not frequently interact with the glycation targets K195 or K199. However, the open form interacts with K199 by forming stable hydrogen bonds, thus supporting the notion that glycation occurs through the open forms of carbohydrates (Pongprayoon and Mori 2018).

Bansode et al. used MD simulations to investigate the structural effects of the binding of tolbutamide—an antidiabetic drug that promotes conformational changes—to bovine serum albumin. The binding caused an increase in the solvent-exposed surface, indicating greater vulnerability to glycation. This was confirmed through in vitro experiments, since an increase in the fluorescence signal linked to AGE formation was observed after tolbutamide binding (Bansode et al. 2015).

REST2 MD simulations were also used to investigate the effect of CEL (Fig. 4) on the structure and dynamics of α-synuclein. CEL formation on all Lys of α-synuclein extended the protein and enhanced its conformational diversity. This enlargement was attributed to the loss of the electrostatic interactions between the N- and C-terminal domains (Ramis et al. 2019). Additionally, it was experimentally proven that CEL inhibited the formation of amyloid fibrils as it stabilized the oligomeric species. In this context, steered MD simulations were conducted on fibril models of the native and glycated α-synuclein, and the average force required to extract the terminal monomer was lower when Lys were modified by CEL (Mariño et al. 2020).

Other computational works on the α-synuclein protein investigated how glycation affected its interaction with other proteins. Semenyuk et al. performed MD simulations to study the interaction of α-synuclein with glyceraldehyde-3-phosphate dehydrogenase (GAPDH). Two models of α-synuclein were created with different degrees of glycation: (i) one exhibited two glycated residues that in vivo undergo SUMOylation; and (ii) the other nine residues were glycated, which, in vivo, undergo ubiquitination. Simulations of GAPDH with the native and glycated α-synuclein variants demonstrated that glycation allows binding outside the anion binding groove. In the native form, the interaction primarily occurred along the C-terminal region of α-synuclein, whereas glycation altered the electrostatics of the N-terminal region and also allowed its binding. Specifically, the model with nine glycated Lys showed many more binding sites on GAPDH, doubling the number of H-bond contacts, salt bridges, and hydrophobic interactions. Indeed, experimental observations indicate that glycation strengthened the interaction of α-synuclein with GAPDH, and caused up to a 40% inactivation of its activity, compared to the 20% inactivation caused by native α-synuclein (Semenyuk et al. 2019).

Subsequently, Sofronova et al. expanded these findings by also considering that glycation can occur on GAPDH and studied its effect on its binding to α-synuclein and RNA. They conducted MD simulations and created two models of glycated GAPDH: (i) one where only the residues in the anion binding groove were glycated; and another in which (ii) glycation was uniform across the entire surface. Glycation significantly hindered the binding to α-synuclein, as the number of H-bonds, electrostatic, and hydrophobic interactions markedly decreased. While in the native form GAPDH interacted with positive residues and α-synuclein through its negatively charged region, the uniformly glycated GAPDH interacted through negatively charged residues, and α-synuclein through positive residues. When the only glycated residues were those in the binding site, the number of electrostatic interactions was reduced to 30% of those observed in the native state. In both glycation models, the binding occurred in different regions of GAPDH, and no preferential binding zone can be concluded. RNA binds to native GAPDH through a positive groove, but also there is a secondary binding site outside the groove. When the positive groove is glycated, RNA binding was only observed in the secondary binding zone. In simulations with uniformly glycated GAPDH, no RNA binding was observed in any case (Sofronova et al. 2021).

Sartore et al. also studied the impact of glycation on the interaction between angiotensin-converting enzyme-2 (ACE2) and the SARS-CoV-2 spike protein. They found a significant loss of interactions between the protein surfaces when Lys were glycated. The number of both polar interactions (H-bonds, salt bridges) and non-polar interactions was reduced by roughly half upon glycation. Consistently, the average interaction distances between proteins increase with the glycation of Lys. These results negate the hypothesis that the interaction between these two proteins is the cause of a higher risk of consequences in diabetic patients due to SARS-CoV-2 (Sartore et al. 2021).

Finally, we would like to mention that computational chemistry has also been employed to study the effect of guanine glycation mediated by GO in DNA models. It was found that as the degree of glycation was increased, the modified nitrogenous bases tend to exit the double helix structure and remain in that altered conformation during the simulations. This supports the hypothesis that they may be unstacked long enough to react with other nucleobases from the opposite strand and form cross-links (Vilanova et al. 2017). Additionally, computational chemistry has also been used to study the glycation of amino phospholipids in membrane models. More precisely, it was found that (i) there was a high likelihood of formation of a Schiff base between phosphatidylethanolamine and acetaldehyde (Solís-Calero et al. 2010, 2012); (ii) the Amadori rearrangement proceeded to a significant extent (Solís-Calero et al. 2013); and (iii) the formation of carboxymethylphosphatidylethanolamine (Solís-Calero et al. 2015), all lead to the destabilization of the ordered lipid membranes, which suggests that glycation is likely to have a harmful effect of the integrity of the biological membranes.

### On the interplay between glycation and aggregation

Besides its effect on the protein structure and function, glycation might also have a dramatic effect on its aggregation propensity. In fact, glycation has been suggested as a potential triggering factor of highly prevalent neurodegenerative diseases, such as Alzheimer’s or Parkinson’s diseases (Miranda and Outeiro 2010; Sirangelo and Iannuzzi 2021; Taghavi et al., 2017). The hallmark of these disorders is the accumulation of amyloid plaques, which are composed of protein aggregates (Chiti and Dobson 2017). As life expectancy rises and diabetes rates increase, the number of people diagnosed with these neurodegenerative diseases has increased (Deuschl et al. 2020). Hence, it is imperative to understand the role of glycation in fostering amyloid aggregation and cytotoxicity to better understand the molecular mechanisms underlying these processes.

Glycation was initially identified as a factor enhancing aggregation propensity, mainly as a result of its chaotropic effect. Numerous studies have provided evidence to support this hypothesis by investigating the effect of glycation on the aggregation of HEWL (Fazili and Naeem 2013; Ghosh et al. 2013), β-amyloid peptide (Chen et al. 2006; Jana et al. 2016), hemoglobin (Iram et al. 2013), albumin (Bouma et al. 2003; Sattarahmady et al. 2007), α-crystallin (Kumar et al. 2004), β2-microglobulin (Kong et al. 2011), human islet amyloid polypeptide (IAPP) (Kapurniotu 2001), or α-synuclein (Chen et al. 2010), among others. Furthermore, other studies have shown that glycation-induced aggregation does not necessarily imply protein unfolding (Adrover et al. 2014; Chen et al. 2009).

Recent investigations have challenged the assumption of an amyloid aggregation tendency enhancement due to glycation. In fact, it seems that glycation reduces the propensity of amyloid fibril formation. For instance, glycation of insulin with MG or d-ribose inhibits amyloid formation but promotes the formation of cytotoxic oligomers (Iannuzzi et al. 2016; Oliveira et al. 2011). However, insulin glycation mediated by glucose either accelerates or strongly inhibits amyloid aggregation, depending on the environmental conditions (Alavi et al. 2013). Similarly, when cytochrome c is modified by MG, it triggers the formation of native-like aggregates without promoting the formation of amyloid fibrils (Oliveira et al. 2013). Glycation of β2-microglobulin (Hashimoto et al. 1999) or superoxide dismutase I (SOD1) (Sirangelo et al. 2016) with glucose inhibits its amyloid fibrillation, and albumin glycated with ribose stabilizes highly cytotoxic oligomeric species instead of promoting amyloid fibril formation (Wei et al. 2009). Ribose also promotes the formation of highly toxic aggregates from α-synuclein, Tau, and β2-microglobulin (Chen et al. 2009, 2010; Kong et al. 2011). Furthermore, cross-linking AGEs might covalently bind monomeric proteins, and this can disrupt their normal folding and, consequently, promote aggregation. For instance, GLA induces the formation of intermolecular crosslinking AGEs on HEWL, which triggers a polymerization cascade that yields the formation of insoluble spherical-like aggregates (Mariño et al. 2017). In addition, changes in the aggregation tendency of a protein can also occur without modifying the protein structure, but changing the overall protein charge or by forming new hydrophobic patches due to its glycation. One example is the glycation of HEWL with ribose, which triggers its native-like aggregation by modifying its surface hydrophobic profile (Adrover et al. 2014). Similarly, Tau glycated with ribose also seems to form cytotoxic globular aggregates without involving rearrangements in the secondary or tertiary structure of the protein (Chen et al. 2009).

The aggregation behavior of amyloid-forming proteins depends on the chemical nature of the glycating agent. While glucose-glycated and CML-modified IAPP promote the formation of amyloid aggregates, glycation of IAPP with MG seems to slow down the aggregation process and alter the aggregate morphology (Hsu et al. 2019; Kapurniotu et al. 1998; Milordini et al. 2020). Moreover, modifications of α-synuclein with MG, GO, or ribose significantly reduce its fibrillization propensity, but stabilize its oligomers and promote the formation of molten globule-like aggregates (Chen et al. 2010; Lee et al. 2009; Padmaraju et al. 2011; Miranda et al. 2017). Nevertheless, the formation of CEL moieties on the 15 Lys of α-synuclein completely inhibits the formation of both amyloid fibrils and soluble oligomeric species (Mariño et al. 2020). Additionally, the formation of CEL on Aβ-peptide, as well as MG glycation, reduce the formation of amyloid fibrils producing stable oligomers (Emendato et al. 2018; Ng et al. 2019).

Nevertheless, the effect of glycation as a promoter of aggregation could go well beyond its effect on the protein itself. Once Lys become glycated, the cellular clearance mechanism mediated by the ubiquitin–proteasome system becomes impaired, because Lys ubiquitination cannot occur, and glycated proteins cannot be degraded by the proteasome. Thus, this process favors the aggregation and the accumulation of protein inclusions (Uceda et al. 2020).

The cytotoxicity of these proteinaceous species appears to depend on the conformational state of the constituent proteins. Regardless of the specific protein involved, all amyloid fibrils share a common archetypal all-β tertiary structure (Fitzpatrick and Saibil 2019). In contrast, oligomers are formed by completely or partially disordered monomers, which then rapidly evolve to form insoluble protofibrils (Liu and Luo 2023). Nevertheless, native folded globular proteins may exhibit some tendency to form aggregated species without undergoing unfolding, simply by populating native-like conformations (Chiti and Dobson 2017). Although both species are accepted to be pathogenic, insoluble fibrillar aggregates are associated to disease progression, while soluble oligomers generally correlate with cellular toxicity (Chiti and Dobson 2017; Kayed and Lasagna-Reeves 2013). Soluble oligomers have been proved to be more toxic than amyloid fibrils in the case of Aβ-peptide (De et al. 2019; Shea et al. 2019), α-synuclein (Guerrero et al., 2013; Winner et al. 2011), apomyoglobin (Iannuzzi et al. 2015) SOD1 (Sirangelo et al. 2016), and insulin (Iannuzzi et al. 2016), among others.

These findings underscore the intricate and multifaceted nature of the impact of glycation on protein behavior, which seems to be strongly dependent on the structural modification induced by the glycating agents. Consequently, protein glycation can exert both promoting and inhibitory effects on the formation of amyloid fibrils and other intermediate species, with varying levels of cytotoxicity depending on the conformation of the constituent protein.

### A direct involvement of glycation in the development of age- and diabetic-related diseases

Inevitably, the final consequences of the glycation of long-lived proteins (e.g., collagen, crystallin, or hemoglobin) are their loss of function and the development of glycation-related diseases, whose prevalence notably increases under hyperglycemic conditions. For instance, the direct involvement of glycation in the pathogenesis of diabetic complications, such as neuropathy, cardiomyopathy, nephropathy, or retinopathy, is well established (e.g., Singh et al. 2001). Additionally, it has also been demonstrated that glycation contributes to the pathology of other disorders such as neurodegenerative diseases, vascular stiffening, atherosclerosis, inflammatory arthritis, and osteoarthritis (Jahan and Choudhary 2015). There are two mechanisms through which AGEs are believed to contribute to aging and to diabetes-related diseases: (i) by binding specific pro-oxidant and pro-inflammatory receptors on cell membrane, mainly the receptor for advanced glycation end products (RAGE), and (ii) through the alteration of protein structure, properties, and function (Sirangelo and Iannuzzi 2021).

RAGEs are the most common receptors to which AGEs bind. They are multi-ligand protein receptors that belong to the immunoglobulin superfamily, and they are present at low levels on the surface of several cell types such as neurons, microglia, brain endothelial cells, or astrocytes (Neeper et al. 1992; Brett et al. 1993). RAGEs contain an extracellular V-type domain that recognizes and binds AGEs, which is followed by two C-type domains, a transmembrane helix, and a C-terminal cytosolic domain that carries out the signal transduction (Xie et al. 2013; Basta et al. 2004). The V-type domain is able to recognize different types of AGEs via their interaction with different regions within the domain. However, all of them are located in the positively charged areas of the domain and they display a significant flexibility, thus able to accommodate ligands of different chemical nature (Xie et al. 2008; Xue et al. 2011; Koch et al. 2010). The AGE-RAGE binding triggers the activation of several intracellular signaling cascades, which leads to the rapid generation of ROS and the production of inflammatory cytokines (Rouhiainen et al. 2013). While limited inflammatory responses can play a role in health, increased glycation might lead to aberrant activation of RAGEs, thus exacerbating the oxidative stress typical of the abovementioned degenerative disorders and contributing to sustained inflammation, potentially leading to cell death.

In any case, protein glycation and the AGE-induced activation of RAGEs induce the development of a broad set of complications that become much more prevalent under DM.

*Cataract formation* is one of the DM-related diseases showing a relatively early presentation. This is because the glucose diffusion into the lens is not insulin-dependent, thus the eye lens is one of body’s organs with a higher propensity to be affected by hyperglycaemia. Diabetic patients have 2–5 times higher risk of developing cataracts than non-diabetic patients, and the disease occurs at an earlier age (Javadi and Zarei-Ghanavati 2008). The accumulation of AGEs, the accelerated polyol pathway, the activation of protein kinase C, and oxidative stress have all been suggested as contributors to diabetic cataracts (Obrosova et al. 2010). In fact, Duhaiman found higher levels of AGEs in cataract of diabetic patients than in that of non-diabetic patients (Duhaiman 1995), thus suggesting that glycation has a clear role in the development of this disease. The accumulation of AGEs can also activate RAGEs in the cells of the eye, thus contributing to the development not only of cataracts, but also age-related macular degeneration and diabetic retinopathy (Kandarakis et al. 2014).

*Diabetic vascular complications* are the main cause of diabetes-related mortality for both DM1 and DM2 subjects (Einarson et al. 2018), and in them, it seems that MG plays a major role (Stratmann 2022). In these diseases, most of the arteries experience damage and medial calcification and, therefore, loss of elasticity and stiffening (Stary 1989). These processes have been correlated with the serum level of pentosidine, which also correlates with arterial thickness in DM2 patients (Yoshida et al. 2005). Collagen, fibronectin, laminin, or elastin, all of them long-life proteins located in the sub-endothelial extracellular matrix, induce tissue rigidity when glycated, thus causing high blood pressure that might result into atherosclerosis and thrombosis (Forbes et al. 2004). The formation of cross-linked AGEs on collagen promotes the loss of elasticity and stiffening of the wall vessel (Birukov et al. 2021). In contrast, the Lys content in elastin is notably poor; thus, it is not affected by glycation, although its functionality can be affected due to its binding to CML (Sell and Monnier 2012). In fact, there is a clear correlation between plasma AGE levels and aortic stiffness (McNulty et al. 2007). The diastolic left ventricular dysfunction is also observed in DM, which stimulates the heart failure. This occurs due to myocardial fibrosis, the deposition of AGEs in the myocardium, and the related hypophosphorylation of the N2B of titin isoform (Falcão-Pires et al. 2011). In addition, glycated proteins also trap nitric oxide, which promotes vasodilation and inhibits various mechanisms involved in the development of atherosclerosis (Xu et al. 2003). Moreover, RAGE-AGE interactions reduce the activity of endothelial nitric oxide synthase, the enzyme that produces nitric oxide (Xu et al. 2003). The synthesis of prostacyclin, a vasodilator, is also slowed by the presence of AGEs, which at the same time increase the synthesis of endothelin-1, a vasoconstrictor (Yamagishi et al., 1998; Quehenberger et al. 2000). Stimulation of RAGEs also triggers a pro-coagulant state due to the reduced activity of thrombomodulin (Esposito et al. 1989), which can turn into thrombosis.

Around 40% of DM1 and DM2 patients are affected by *nephropathy*, which is characterized by a progressive dysfunction of glomerular filtration (Chiarelli et al. 2000). There are several risk factors for diabetes-induced kidney failure, but it seems that renal accumulation of AGEs (mainly CML and pentosidine) is responsible for the development of diabetic nephropathy (Vlassara et al. 1994). Glycation of renal proteins induces the accumulation of proteolysis-resistant species, which build up in the glomerular basement membrane (Forbes et al. 2003). However, this process is also stimulated by AGE-RAGE interactions, which trigger the synthesis of collagens (type I, III, and IV) and fibronectin by renal cells (Pastino et al. 2017). In addition, the expression of the anti-fibrotic agent, bone morphogenetic protein-7, decreases in the kidneys of DM patients. Hence, the accumulation of AGEs becomes toxic for the kidneys, as they gradually induce a reduction in their filtration capacity. As a consequence, AGEs cannot be eliminated and accumulate in the body (Makita et al. 1991).

Another diabetic-related pathology where glycation seems to play a crucial role is in the development of *neuropathy* (Thornalley 2022). The different types of neuropathies are characterized by either a loss of sensitivity, paraesthesia (the sensation of tingling), and dysesthesia (abnormal contact sensations), which can be accompanied by burning pain. All these conditions lead to a loss of nociception and to the appearance of injuries as a result of the patient’s decreased perception, whose appearance is linked to a healing deficit and to the appearance of a pathologic condition known as “diabetic foot,” which often ends with amputation (Said 2013; Duran-Jimenez et al. 2009). All this appears because the capillaries that perfuse nerves are affected by AGEs (Sugimoto et al. 2008). Extracellular glycation thickens the basement membrane and increases parietal permeability by changing its electrical charge. In addition, endothelial RAGEs are activated in perineurial and endoneurial blood vessels, which leads to vascular malfunction. All of these processes lead to circulatory issues in the capillaries and the onset of hypoxia, which affects nervous tissue. Glycation has also a direct effect on the immune system, which enhances the possibility of skin wounds and infections (Barwick et al. 2016).

The *mass and function of skeletal muscles* are also affected by the accumulation of AGEs. It seems that myosin structure and the interaction between actin and myosin can be modified as a result of the formation of AGEs on them (Snow et al. 2007). The increase in the crossed-linked collagen and pentosidine was found to be inversely proportional to the muscle wet weight (Haus et al. 2007).

Glycation also affects the rate of *skin aging*, yet the mechanisms involved in this process have not been identified. The accumulation rate of glycated collagen is estimated to be about 3.7% per year, although lifestyle, exposure to UV light, and diet affect this percentage (Danby 2010). With aging, the skin tends to be less elastic, thin, and dry. These processes tend to be accelerated by exposure to UV irradiation, tobacco, pollution (Gkogkolou and Böhm 2012), and an enhanced glycation process. Pentosidine causes skin inflammation (Ichihashi et al. 2011) and glycation of keratin changes its transparency (Yonei et al. 2015) due to AGE accumulation in the dermis. Prolonged exposure to sunlight could speed up the formation and accumulation of CML, thus causing an abnormal elastin accumulation in the dermis (Mizutari et al. 1997).

AGE accumulation has also been associated with a higher propensity to develop neurodegenerative diseases. One of them is *Alzheimer’s disease* (AD), the most common type of dementia, which has a large incidence in people of age over 65 (Ferri et al. 2005). Several risk factors have been associated with AD including genetics, age, head trauma, hypertension, diabetes, and high cholesterol (Burns and Iliffe 2009). AD has also been correlated with glycation. Several evidences demonstrate that AGEs accumulate in senile plaques and neurofibrillary tangles isolated from AD brains with AGEs were found in the Tau and Aβ aggregates (Sasaki et al., 1998; Lüth et al. 2005).

*Parkinson’s disease* (PD) is another very common neurodegenerative disease that seems to be stimulated by glycation. In fact, diabetic people have a ~ 38% higher propensity to develop PD than non-diabetic people (Yue et al. 2016). PD is characterized by resting tremors, rigidity, slow movements, and postural instability. PD starts with the degeneration of dopaminergic neurons in the *substantia nigra* of the midbrain, where dopamine is mainly produced (Guerrero et al., 2013). AGE formation was reported in the Lewy bodies (LBs) isolated from *substantia nigra* (Miranda and Outeiro 2010). In addition, AGEs were found in cerebral cortex and amygdala, regions that also overexpress RAGEs. AGEs were mainly co-localized on aggregated α-synuclein, the main component of LBs, so it was thought that they stimulate its aggregation (Padmaraju et al. 2011). α-Synuclein has 15 Lys residues able to be glycated. This is highly important in a pathological context, since α-synuclein oligomers are highly toxic (Guerrero et al., 2013), as they disrupt cellular membrane and alter their permeability. Moreover, monomeric and oligomeric glycated α-synuclein generate reactive oxidative species, thus increasing oxidative stress. Finally, glycated oligomers are resistant to proteolysis, thus causing proteasome dysfunction (Miranda et al. 2017). The cells with proteasome dysfunction proceed to autophagy and are eliminated. Glycated α-synuclein activates microglia and causes neuroinflammation, but also interacts with RAGEs triggering the release of NF-kB. Since NF-kB also regulates RAGE expression, its overexpression causes more AGE binding and, therefore, it forms a feedback loop that activates the inflammatory pathway and the neuronal death (Guerrero et al., 2013). In addition, the work of our group has shown that the formation of CEL on α-synuclein inhibits one of its most important biological functions, i.e., its ability to bind and cluster synaptic vesicles carrying dopamine, thus hampering correct neurotransmission (Uceda et al. 2022).

*Prion diseases* are another type of fatal neurodegenerative disease that starts with the misfolding of the cellular prion protein (PrP^C^)—normally involved in signal transduction (Didonna 2013)—which in the disease state instead aggregates and accumulates. The process of aggregation is accompanied by neuronal loss and spongiform alterations, which are the characteristics features of Creutzfeldt-Jakob disease (CJD) (Knight and Will 2004), a disease that is both sporadic, genetic, and transmissible in nature (Prusiner 1998). The “protein only” hypothesis states that CJD is caused by the conversion of natively folded PrP^C^ into non-native state, which is resistant to degradation by proteinase K (PrP^res^) (Asher and Gregori 2018). Use of anti-AGEs and anti-RAGE antibodies has allowed for the direct observation of AGEs and RAGEs in the development of CJD (Sasaki et al. 2002). These studies revealed a co-localization of PrP-positive granule AGEs and RAGE. Glycation of PrP^C^ was observed to occur on K23, K24, K27, and R37 (Choi et al. 2004), which results into a PrP^C^ resistant to protease degradation, thus being a powerful inductor of oxidative stress. Thus, glycation might play a key role in the pathogenesis of prion diseases since it would protect PrP against degradation.

DNA glycation has been shown to promote mutagenesis and stimulate the development of *cancer.* This occurs because AGEs stimulate RAGEs, thus activating several molecular signaling pathways such as the PI3K/AKT, JAK/STAT, NF-κB, Ras/MAPK, Rac1/cdc42, p44/p42, p38, or the SAP/JNK/MAPK pathways (Palanissami and Paul 2018). These inflammatory processes also induce epigenetic changes in pre-malignant lesions and silence tumor suppressor genes (Palanissami and Paul 2018). In addition, AGEs-RAGE interactions also activate NADPH oxidases, thus causing increased intracellular oxidative stress. The expression of RAGEs is usually upregulated in most types of cancers, such as colorectal (Azizian-Farsani et al. 2020), pancreatic (Swami et al. 2020), prostate (Akkus et al. 2021), lung (Chen et al. 2020), or breast (Zhang et al. 2020) cancers.

More recently, it has been shown that accumulation of AGEs could be a potential risk factor for increased *COVID-19-linked fatalities* in elderly patients (Sellegounder et al. 2021). RAGEs expressed by DM2 epithelial cells in the alveolar sac have been reported to be associated with lung inflammation caused by COVID-19 (Wang et al. 2020).

### Endogenous mechanisms of detoxification against glycation

**Fig. 6:**
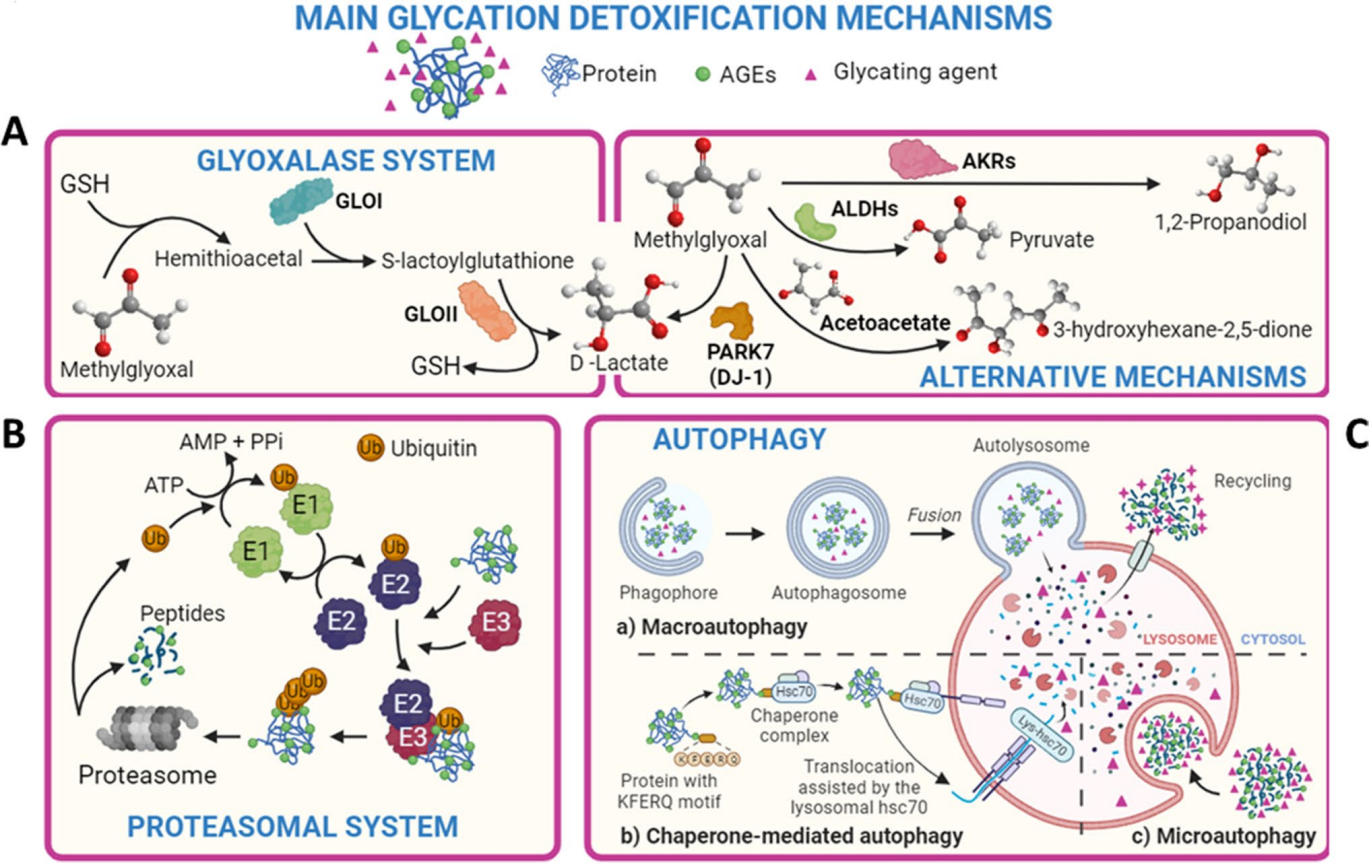
Main endogenous mechanisms of detoxification against glycation. The glyoxalase system (**A**) plays a crucial role in degrading reactive dicarbonyl compounds, which serve as glycating agents. In addition, complementary alternative mechanisms (*right*) further contribute to the elimination of these harmful compounds. The proteasomal system (**B**) and autophagy (**C**) are mechanisms responsible for breaking down glycated proteins with compromised functionality or structure

Given the pathological effects that glycation has on the normal function of the body, evolution has designed specific mechanisms to reduce glycation events and also to eliminate and deglycate biomacromolecules (Fig. 6). One of the most studied mechanisms is the *glyoxalase system* that consists of two cooperating enzymes, i.e., Glo-1 and Glo-2, that rapidly degrade free MG, GO, and 3-DG before they can react with biomolecules (Aragonès et al. 2021; Kold-Christensen and Johannsen 2020). This detoxifying mechanism has a high specificity for MG (Thornalley 1990), which rapidly reacts with glutathione to form the corresponding hemithioacetal, that is the specific substrate of Glo-1. Subsequently, Glo-1 converts it into S-d-lactoylglutathione, that is then hydrolyzed into d-lactate by Glo-2 (Fig. 6A) (Aragonès et al. 2021; Shinohara et al. 1998). This system is thought to be a key component in the maintenance of the intracellular levels of reactive dicarbonyls (Nigro et al. 2017; Thornalley 2003a).

There are alternative routes to the glyoxalase system for the detoxification of dicarbonyl compounds (Fig. 6A) (Aragonès et al. 2021; Kold-Christensen and Johannsen 2020). These systems assist the glyoxalase pathway to detoxify AGEs, but their physiological relevance remains unclear. One of these secondary mechanisms is carried out by the DJ-1 protein, which converts MG into lactate in the absence of glutathione (Matsuda et al. 2017). In addition, it has been suggested that DJ-1 can also repair MG-modified nucleotides and proteins at the early glycation stages (Lee et al. 2012; Richarme et al. 2015), although other works suggest the opposite (Mazza et al. 2022). Another mechanism is carried by aldo–keto reductases, a set of enzymes that reduce ketones and aldehydes into primary and secondary alcohols (Baba et al. 2009; Vander Jagt et al. 2001). Aldo–keto reductases tend to protect against MG damage (Li and Ellis 2014) since their inhibition increases the MG levels (Baba et al. 2009). Moreover, aldehyde dehydrogenases, whose expression is boosted by MG (Morgenstern et al., 2020), are also able to oxidize MG to pyruvate (Vander Jagt and Hunsaker 2003). In addition, aldehyde dehydrogenase activities are increased as a result of Glo-1 knockout to compensate for the lack of the glyoxalase system (Lodd et al. 2019). In any case, it seems that some of these alternative routes are highly tissue-dependent (Schumacher et al. 2018), and they might produce harmful compounds like γ-diketones, which have been linked with different health problems (Spencer and Chen 2021).

In addition to all these detoxifying mechanisms, cells also have proteolytic systems responsible for breaking down glycated proteins. The ubiquitin–proteasome system (UPS) plays essential roles in degrading misfolded or glycated proteins, thus preventing the accumulation of AGEs and reducing their harmful consequences (Goldberg 2003; Taylor 2012; Uchiki et al. 2012). In the UPS system, ubiquitin molecules are attached to target proteins, marking them for degradation by the proteasome (Fig. 6B) (Goldberg 2003). However, glycation of Lys residues impairs the covalent binding of ubiquitin to these residues; thus, glycated proteins tend to become resistant to proteasomal degradation and start to aggregate. These large aggregates turn out to be inaccessible to proteasomal proteases, so they are stored in the aggresome and are then degraded via macroautophagy (Fig. 6C) (Takahashi et al. 2017). This process involves the sequestration and degradation of cellular components in a double-membrane structure called the autophagosome, which fuses with lysosomes leading to the degradation of the aggregates (Takahashi et al. 2017; Zientara-Rytter and Subramani 2019). Additionally, microautophagy and chaperone-mediated autophagy also promote degradation of cytoplasmic components in the lysosome, such are glycated proteins (Gómez et al. 2021) (Fig. 6C). Aging and excessive glycation levels have been shown to negatively impact UPS due to direct glycation of their components (Aragonès et al. 2020; Queisser et al. 2010).

Other enzymes such as fructosamine 3-kinase, fructoselysine oxidase, or fructoselysine 6-kinase have also been proved to deglycate proteins (Delpierre et al. 2000; Szwergold et al. 2001; Takahashi et al. 1997; Wiame et al. 2002). The mechanisms of action of some of these enzymes have been investigated using theoretical calculations and computer simulations, such as in the studies published by Rigoldi et al. who investigated the interaction of five different fructosyl amino acid oxidases (amadoriase I, amadoriase II, FPOX-E, N1-1-FAOD, and PnFPOX) with fructosyl-lysine (f-Lys) and fructosyl-valine (f-Val) to understand the specificity of these enzymes (Rigoldi et al. 2016). The structures of the five enzymes are very similar, and their main differences are in the loops defining the entrance to the active site tunnel. They analyzed the sizes of cavities and access tunnels to the active site, as well as the polarity of residues along the tunnel, to investigate the affinity of the active site for f-Lys or f-Val. The simulations suggested that amadoriase I, amadoriase II, and FPOX-E possess a better environment to stabilize f-Lys (Rigoldi et al. 2016).

In addition to these cytoplasmatic processes, other detoxification processes also occur simultaneously in other cellular locations. Heat-shock proteins (Hsps), which are molecular chaperones that facilitate the folding of proteins or target misfolded proteins for clearance, also help to maintain cellular health and function (Sudnitsyna and Gusev 2015). Therefore, increased levels of Hsp27 suppressed the detrimental effects induced by MG on α-synuclein, also reducing MG-induced α-synuclein aggregation in cells (Miranda et al. 2020).

### Types and effectiveness of glycation inhibitors: current state of pharmaceutical treatment

Although the body has developed systems to protect against glycation and its pathogenic consequences, in some situations, such as DM, their action is not sufficient to prevent diabetes-induced diseases. Hence, the pharmaceutical industry has developed a set of drugs whose mechanism of action is to prevent the glycation process or revert its consequences. In the recent years, an important number of review articles covering various aspects of glycation inhibition have been published, and interested readers are encouraged to consult the following references for a deeper insight (Abbas et al. 2016; Anwar et al. 2021; Jahan and Choudhary 2015; Jud and Sourij 2019; Nenna et al. 2015; Peng et al. 2011; Peyroux and Sternberg 2006; Rahbar and Figarola 2002; Salazar et al. 2021; Sarmah and Roy 2022; Song et al. 2021; Sourris et al. 2009; Yamagishi et al. 2008; Younus and Anwar 2016). Among these articles, Yamagishi et al. (2008), Sourris et al. (2009), and Nenna et al. (2015) emphasize how glycation inhibitors can prevent diabetic-induced cardiovascular complications. The articles by Nenna et al. (2015), Jud and Sourij (2019), and Salazar et al. (2021) provide data from various clinical studies conducted with different AGE inhibitors. Jahan and Choudhary (2015) also compile patents registered up to 2014 related to AGE inhibitors. Among the more recent articles, a distinction is made between synthetic and naturally derived inhibitors, highlighting that the latter may be interesting for treatment, since they generally exhibit lower toxicity and are more economical (Abbas et al. 2016; Anwar et al. 2021; Jahan and Choudhary 2015; Peng et al. 2011; Sarmah and Roy 2022; Song et al. 2021; Younus and Anwar 2016).

Given the diversity of possible reaction pathways involved the formation of AGEs, glycation inhibitors usually exhibit several mechanisms of action. According to Khalifa et al.’s (1999) original classification, there are six types of inhibitors (Rahbar and Figarola 2002) with these being as follows:

A. Molecules that compete with carbohydrates to react with free amino groups of biomolecules. In practice, this does not present a viable therapeutic strategy since it is not feasible to block all amino groups in the organism. Examples include pyridoxal-5′-phosphate or aspirin.
B. Molecules that react with aldoses and ketoses, preventing them from undergoing protein glycation. These inhibitors can also interact with species containing carbonyl groups generated in later stages of glycation, such as Amadori products. These inhibitors pose the risk of deactivating species with important carbonyl groups for the organism, such as pyridoxal-5′-phosphate. Aminoguanidine and pyridoxamine are examples of these inhibitors.
C. Molecules that interfere on side reactions along the glycation process. These are metal chelators and antioxidants. Both types of inhibitors eliminate species that directly or indirectly participate, promote, or accelerate oxidation reactions that generate ROS and AGEs. Examples of metal chelators include pyridoxamine and phytate, and antioxidants include vitamins C and E.
D. Molecules capable of trapping highly reactive dicarbonyls like GO or GLA. Diabetic patients may exhibit high concentrations of these species due to metabolic imbalances (Khalifah et al. 1999). Aminoguanidine also falls into this category.
E. Species capable of blocking Amadori products and preventing their conversion into AGEs. Aminoguanidine (Khalifah et al. 1999) and pyridoxamine (Voziyan et al. 2002) can act in this manner.
F. Molecules acting as AGE breakers, which can revert crosslinking AGEs but do not prevent their formation. ALT-711 has been suggested to act as an AGE breaker since it seems able to reverse diabetes-induced increase of artery stiffness (Wolffenbuttel et al. 1998) and has a renoprotective effect (Coughlan et al. 2007)

Next, we highlight key chemical features of inhibitors of glycation.

**Fig. 7:**
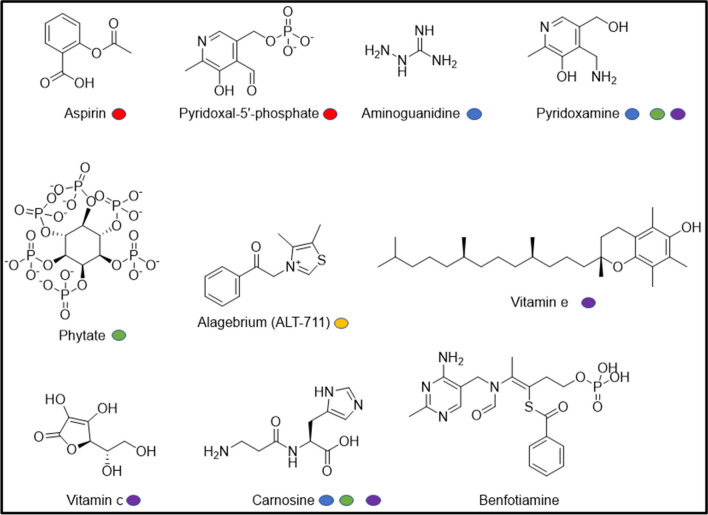
Chemical structures of the most relevant inhibitors of glycation. The compounds labeled with a red circle (  ) are those that can inhibit glycation through the protection of the amino groups that are prompt to be glycated. The compounds labeled with a blue circle (  ) are those that can inhibit glycation via scavenging carbonyl compounds. The compounds labeled with a green circle (  ) are those that can inhibit glycation via chelating metal cations that promote it. The compounds labeled with a purple circle (  ) are those that can inhibit glycation via the neutralization of ROS. The compounds labeled with a yellow circle (  ) are those that can act as AGE breakers

*Aminoguanidine* acts as a carbonyl scavenger (Fig. 7), mainly dicarbonyl species, thus preventing the formation of the initial glycation products, hindering the evolution of Amadori compound into AGEs, and averting the formation of crosslinking AGEs (Thornalley 2003b). Aminoguanidine may delay complications arising from diabetic neuropathy, nephropathy, and retinopathy (Jud and Sourij 2019; Peyroux and Sternberg 2006; Rahbar and Figarola 2002). Two phase III clinical trials were conducted with aminoguanidine. One included DM1 patients, for which a slower progression of retinopathy was observed. The second study included DM2 patients, but it was stopped due to apparent lack of results and adverse side effects on liver function (Freedman et al. 1999; Jud and Sourij 2019; Nenna et al. 2015; Sourris et al. 2009).

*Pyridoxamine* is one form of vitamin B6 that is able to inhibit glycation (Fig. 7). Its main mechanism of action involves the chelation of metals with redox capacity to oxidize the Amadori product (Voziyan et al. 2003), but it is also able to scavenge reactive carbonyl intermediates (Voziyan et al. 2002). Toxicity studies indicated that pyridoxamine is safe (Voziyan and Hudson 2005).

*Carnosine* is the β-alanine-histidine dipeptide and it is a natural molecule present in all organisms (Fig. 7). It exhibits various mechanisms of inhibition. It can react with sugars to prevent the formation of AGEs, and it can react with proteins that had been previously modified by MG through its reaction on the second carbonyl group, thus preventing the formation of crosslinking AGEs (Alhamdani et al. 2007; Hipkiss 2000). Carnosine can chelate metal ions (Price et al. 2001) and it can scavenge ROS (Klebanov et al. 1998). Additionally, the AGEs with which carnosine reacts, cease to be recognized by RAGEs (Hipkiss 2000). Carnosine was reported to have a protective effect in nephropathy models (Janssen et al. 2005).

*Benfotiamine* is a derivative of thiamine (vitamin B1; Fig. 7) that acts as a potent activator of transketolase with the ability to reduce the levels of AGEs. However, the results in clinical trials were moderately successful. One did not show improvement in peripheral nerve function (Fraser et al. 2012), whereas the other caused a decrease in the albuminuria (Rabbani et al. 2009), although such an effect was not observed in another study with a larger sample of patients (Alkhalaf et al. 2010). Another clinical trial indicated that dietary supplementation with benfotiamine reduced AGE levels in DM2 patients with a high AGE diet (Stirban et al. 2006).

Another way to inhibit the glycation involves avoiding the RAGE cascade that increases oxidative stress and, thereby, promotes the formation of more AGEs. This can be achieved by eliminating free AGEs, blocking the interaction of AGEs with the RAGE receptor, and blocking the RAGE activation cascade (Kim et al. 2005; Peyroux and Sternberg 2006).

### Summary and perspectives

Protein glycosylation and glycation are distinct processes involving carbohydrate modification of proteins. Glycosylation, mediated by enzymes, attaches polysaccharides to proteins during biosynthesis, influencing protein folding and function. In contrast, glycation involves non-enzymatic attachment of glucose to proteins, and this occurs randomly in the intra- and extracellular spaces. This ends with the formation of AGEs, which are implicated in DM-related diseases, as well as in other aging-related pathologies. The intracellular glycolysis produces MG, which further contribute the AGE formation and cell damage. Dysregulated glycation, exacerbated in conditions like DM, leads to AGE accumulation, affecting cellular structures and functions.

Protein glycation initiates with the reaction between protein amino groups and glucose, forming Schiff bases that rearrange into Amadori products. These products can further evolve into AGEs. Concurrently, sugar moieties can undergo fragmentation reactions, producing 3-DG and MG. These RCS contribute significantly to glycation, forming diverse AGEs. Glycation propensity varies among different sugars, being glucose and ribose those with higher glycation relevance in vivo. Glycation primarily targets Lys and Arg, but RCS also modify Cys in reducing environments. Environmental factors such as pH, temperature, and buffer ions influence glycation extent. Additionally, the protein’s half-life affects glycation, with longer-lived proteins accumulating more AGEs. Dysregulation of RCS production leads to carbonyl stress, impacting cellular function. Understanding these mechanisms is crucial for mitigating glycation-related damage. Glycation affects nucleic acids, leading to mutations, and disease like diabetes and cancer. Amino phospholipids, found in cell membranes, become also glycated under hyperglycemic conditions, impacting cell integrity and contributing to diabetic complications and age-related dysfunctions.

For a long time, it was thought that glycation induced structural chaotropic effects and protein unfolding. These assumptions were mainly based on the data obtained using low-resolution techniques. However, high-resolution techniques such as NMR and X-ray diffraction have consistently shown no significant alterations in the secondary or tertiary structure of glycated proteins. This discrepancy led to investigations into the quenching effect of AGEs on protein fluorescence, revealing that AGEs can indeed quench intrinsic fluorescence. Misinterpretations of fluorescence data in the study of glycated proteins have been found, emphasizing the need for caution and the integration of multiple techniques to accurately assess the effect of glycation on protein structure. The limited effect hat glycation has on protein structure can be rationalized by the solvent-exposed nature of Lys and Arg residues, which typically lack crucial long-range contacts defining protein tertiary structure, unless involved in salt bridge interactions.

Glycation disrupts protein function independently of any chaotropic effect on protein structure. AGEs alter protein residues, potentially creating hydrophobic patches that induce aggregation, modifying equilibria between folded and unfolded states, and altering interactions with binding partners or enzymatic activities. Various proteins, including serum albumin, cytochrome c, and myoglobin, exhibit modified functions upon glycation, influencing cytotoxicity, apoptosis induction, or enzymatic activity. Additionally, glycation contributes to oxidative stress, generating ROS and impairing antioxidant capacity. The effects of glycation on protein function are multifaceted and dependent on the specific protein, extent of glycation, and cellular environment, highlighting its complexity and importance in disease pathogenesis.

Computational chemistry has emerged as a powerful tool to better understand the impact of glycation on protein structure and function. MD simulations have been carried out to elucidate glycation effects on proteins like hemoglobin, collagen, and insulin. These simulations reveal altered binding sites, structural changes, and disrupted interactions, shedding light on disease mechanisms. For instance, glycation hinders insulin binding to its receptor and weakens protein–protein interactions, such as between α-synuclein and GAPDH. Additionally, computational chemistry has been used to study the effect of glycation on protein–ligand interactions, as seen BSA-tolbutamide binding. Computational chemistry also extends to studying DNA and lipid glycation, providing insights into cellular dysfunction and membrane destabilization. Overall, computational approaches offer valuable insights into glycation’s molecular mechanisms, aiding in understanding its implications in disease and cellular processes.

Protein glycation might profoundly influence the protein aggregation propensity. While initially thought to enhance aggregation via chaotropic effects, recent studies reveal a nuanced relationship. Glycation can inhibit fibril formation but promote cytotoxic oligomer generation, with outcomes varying by glycating agent and protein type. Additionally, glycation can disrupt protein clearance mechanisms, favoring aggregation and inclusion formation. The cytotoxicity of resulting proteinaceous species depends on their conformational state, with soluble oligomers generally correlating with cellular toxicity. Insoluble fibrillar aggregates are associated with disease progression. Overall, the impact of glycation on protein aggregation is complex and it depends on structural modifications induced by glycating agents, exerting both promoting and inhibitory effects on amyloid fibril formation and cytotoxicity.

The effect of glycation on the protein function, structure, and aggregation might end with the development of pathological events. For instance, glycation of long-lived proteins like collagen or hemoglobin leads to loss of function and contributes significantly to the development of glycation-related diseases, which become more prevalent in diabetic people. AGEs interact with RAGEs triggering oxidative stress, inflammation, and tissue damage. This process is implicated in various complications of diabetes, including neuropathy, cardiomyopathy, nephropathy, and retinopathy. Additionally, glycation plays a crucial role in the pathogenesis of other diabetes-induced disorders like cataracts, vascular stiffening, atherosclerosis, inflammatory arthritis, osteoarthritis, and neurodegenerative diseases such as Alzheimer’s and Parkinson’s. Moreover, glycation contributes to muscle dysfunction, skin aging, and cancer development by promoting mutagenesis and activating inflammatory pathways.

As a result of the harmful effects of glycation, the evolution that has developed several endogenous mechanisms to counteract the detrimental effects of glycation. The glyoxalase system efficiently degrades RCS like MG. Alternative pathways involving DJ-1, aldo–keto reductases, or aldehyde dehydrogenases also contribute to detoxifying RCS. Additionally, proteolytic systems like the UPS degrade glycated proteins to prevent their accumulation. However, glycation of Lys impairs ubiquitin binding, leading to protein aggregation. These aggregates are then degraded via macroautophagy, microautophagy, and chaperone-mediated autophagy. Aging and excessive glycation levels can compromise the efficiency of these detoxification mechanisms. Hsps further aid in maintaining cellular health by facilitating protein folding and clearing misfolded proteins, thereby mitigating the harmful effects of glycation on cellular function and integrity.

Pharmaceutical interventions targeting glycation aim to mitigate the pathological consequences associated with conditions like DM. Various classes of glycation inhibitors have been developed, including those that compete with carbohydrates for amino groups, react with aldoses and ketoses, interfere with side reactions, trap RCS, or act as AGE breakers. Examples of such inhibitors include aminoguanidine, pyridoxamine, carnosine, and benfotiamine, each exhibiting distinct mechanisms of action and potential therapeutic benefits. Clinical trials have demonstrated varying degrees of success, with some inhibitors showing promise in delaying or ameliorating diabetic complications like neuropathy and nephropathy. Additionally, targeting the RAGE cascade offers another approach to inhibit glycation-mediated pathology by blocking AGE-RAGE interactions and downstream signaling pathways associated with oxidative stress and inflammation.

Despite all what we have reported here, there are still many key questions that remain unanswered to fully comprehend the effect of glycation on the development of diabetes-induced disease. Some of them are as follows:

A. *The role of specific glycation products*: While AGEs are implicated in various diseases, the specific contributions of individual glycation products to disease pathogenesis are not fully elucidated. Understanding the relative importance of different AGEs in driving specific pathological processes could inform targeted therapeutic strategies.
B. *Clarify the precise impact of glycation on protein function*: Although it is known that glycation could impair protein structure and function, the precise molecular mechanisms underlying these effects remain unclear. Further investigation is needed to determine how glycation-induced modifications alter protein activity, stability, and interactions with other molecules.
C. *Tissue-specific effects of glycation*: Glycation can occur in different tissues and organs throughout the body, but the impact of glycation on tissue-specific pathologies is not well-defined. Investigating how glycation contributes to disease development in specific tissues could reveal novel therapeutic targets and diagnostic markers.
D. *Interplay between glycation and other post-translational modifications*: Proteins undergo a variety of post-translational modifications, including glycation, phosphorylation, acetylation, and methylation. Understanding how these modifications interact and influence the protein function and cellular signaling pathways is an unexplored area of research.
E. *Development of glycation inhibitors*: While several glycation inhibitors have been identified and investigated, their clinical efficacy and safety profiles are not fully established. Further research is needed to optimize the design and development of glycation inhibitors for therapeutic use in diseases associated with protein glycation.
F. *Impact of glycation on aging*: Glycation has been implicated in the aging process and age-related diseases, but the underlying mechanisms linking glycation to aging phenotypes are not fully understood. Elucidating the molecular pathways through which glycation contributes to aging could provide insights into strategies for promoting healthy aging and preventing age-related diseases.

Only addressing these (but not only) open questions will advance the understanding of the role of protein glycation in health and disease, paving the way for the development of novel therapeutic interventions and diagnostic approaches.

## Data Availability

No datasets were generated or analysed during the current study.

## Materials availabilty

No new materias were presented in this review.
